# Biomarkers in NHL

**DOI:** 10.18632/oncotarget.252

**Published:** 2011-04-01

**Authors:** Sami N. Malek

**Affiliations:** Department of Internal Medicine, Division of Hematology and Oncology, University of Michigan, 1500 E. Medical Center Drive, Ann Arbor, MI 48109

Patients with non-Hodgkin's lymphoma (NHL) are in need of better predictive and prognostic markers; this to be able to more accurately inform them in the clinical setting about their likely future and to better inform clinicians and clinician-researchers about treatment decisions. One of the major impediments to the development of such markers in NHL has been the relative lack of access to well-preserved tumor tissue as the bulk of these cancers reside in secondary lymphoid organs. Procurement of tissue from affected lymph nodes for molecular risk stratification only has not yet been adopted as standard clinical practice.

One of the ways a biomarker with clinical value in NHL could be developed is through analysis of shed tumor products in plasma or serum from patients carrying NHLs. This is the approach taken by He et al., 2011 in work reported in this issue of Oncotarget [1]. The authors began by developing a clever custom-array-based method to capture and enrich for immunoglobulin heavy chain gene fragments contained in sheared DNA isolated from NHL patients followed by use of massively parallel sequencing to generate a large collection of Ig sequences. Given that tumors usually carry one or two rearranged and often somatically hypermutated Ig allele, the identification of these tumor-specific alleles was possible.

The authors demonstrate that they could identify the rearranged Ig genes in primary human NHL-derived DNA and also in paired DNA derived from patient's plasma. They went on to show that they could amplify rearranged Ig fragments from an additional set of NHL patients using just plasma as a source of DNA. To support the analysis of the large collection of DNA sequence tags generated from each sample they developed a set of novel software algorithms for Ig gene identification. In summary, in the majority of patients analyzed the authors were able to identify the tumor-derived Ig sequences and also to enumerate the frequency of these in the input DNA.

One of the most important questions related to this innovative work is whether the approach chosen has identified a tumor mass marker in patients with NHL and whether such a features can be clinically exploited. To answer this question, the further development of this potential new biomarker will need to proceed in an organized and logical manner to fully explore its ultimate potential. First, it will be necessary to conduct a new study using a larger collection of paired patient samples (primary tumor and paired plasma) to generate data on the sensitivity and specificity for plasma-derived DNA testing referenced against tumor DNA as the gold standard. Tests in large cohorts of individuals without known NHL may also be needed to obtain estimates on false positive calls.

Next, and possibly within the same trial, initial estimates for this marker as a tumor mass marker should be obtained. Here, development will be complicated by the absence of validated test that accurately measure total body tumor burden in NHL. Nonetheless, a study correlating results from serial cross-sectional imaging with normalized numbers of tumor specific Ig-tags could be designed. For such a study it would be important to also perform the DNA test serially past completion of the induction chemotherapy program to obtain estimates on the temporal relation of CT findings and Ig-DNA levels. Somewhat complicating such an analysis is the current uncertainty as to what lymphoma cells (live cells or dead cells) actually contribute to and in what proportion to the plasma-detectable DNA.

Future detailed clinical development of this marker would probably best be done within specific NHL subtypes and targeted to clinical situations in which current tumor mass assessments are semi quantitative at best. Within the setting of B-cell-derived NHLs, a number of clinical applications could be envisioned:

Diffuse large B-cell lymphoma (DLBCL), the most common NHL subtypes is treated relatively uniformly using the R-CHOP regimen as front-line therapy. Currently, only approximately 45-50% of patients are cured. While further risk stratification using either clinical tools (international prognostic index) or imaging tools (like PET-CTs performed during or after completion of planned therapy) can be accomplished, one wonders whether serial quantitative IgH DNA analysis as described here could be used to predict which patients are cured and which subset will relapse.

A similar scenario can be identified in mantle cell lymphoma (MCL) for which intensified regimens like the NORDIC regimen can achieve remission-free states and possible cures in a substantial subset of cases. Could plasma-based IgH DNA analysis performed after the completion of the chemo-auto-Tx protocol be used to identify long term survivors?

Finally, with the recent resurgence of maintenance therapy approaches in follicular lymphoma (FL), could IgH DNA analysis be used to identify patients that may most benefit from such an intervention?

In summary, the elegant pilot study by He et al [1], has opened the door to future improved NHL care and we await anxiously expeditious testing of their approach in the clinical setting as outlined above.
